# *SaeRS* Gene Expression Following Exposure to Olive Leaf–Mediated Zinc Oxide Nanoparticles in Multidrug‐Resistant *Staphylococcus aureus*

**DOI:** 10.1155/vmi/4840079

**Published:** 2026-10-27

**Authors:** Ali Haider Alsakini, Munim Radwan Ali, Asmaa Samir Mohsin, Anfal Mohammed Khudhair

**Affiliations:** ^1^ Department of Biology College of Science Mustansiriyah University Baghdad Iraq uomustansiriyah.edu.iq; ^2^ Department of Microbiology College of Medicine Al Iraqia University Baghdad Iraq medcol.mw

**Keywords:** gene expression, green synthesis, multidrug resistance, SaeRS system, *Staphylococcus aureus*, zinc oxide nanoparticles

## Abstract

**Aim:**

This pilot study assessed the transcriptional responses of *saeR* and *saeS* to green‐synthesized zinc oxide nanoparticles (ZnO NPs) prepared using *Olea europaea* leaf extract in four multidrug‐resistant clinical isolates of *Staphylococcus aureus*. Antimicrobial susceptibility profiles and the prevalence of *pvl*, *agrI*, *agrII*, and *agrIII* were also determined.

**Methods and Results:**

Antimicrobial susceptibility testing was performed on 40 clinical isolates. Four multidrug‐resistant isolates were exposed to ZnO NPs at a subinhibitory concentration (1/2 MIC), and *saeR* and *saeS* expression was quantified by RT‐qPCR using 16S rRNA for normalization and paired comparative Ct calculations. Untreated and exposed ΔCt values from the same isolate were compared using exact two‐sided Wilcoxon signed‐rank tests. Resistance was highest to oxacillin (85.0%), fusidic acid (82.5%), ceftaroline (72.5%), and rifampin (70.0%), whereas gentamicin, doxycycline, tigecycline, and linezolid showed 97.5% susceptibility. The *pvl* gene was detected in 27.5% of isolates; *agrI*, *agrII*, and *agrIII* were detected in 17.5%, 20.0%, and 15.0%, respectively. *saeR* expression decreased numerically in all four isolates, but the paired comparison was not statistically significant (*p* = 0.125). *saeS* expression decreased in three isolates and increased in one isolate; this paired comparison was also not significant (*p* = 0.250).

**Conclusions, Significance and Impact:**

The findings provide descriptive, isolate‐specific evidence of transcriptional responses of saeR and saeS to sub‐MIC ZnO‐NP exposure in the four MDR S. aureus isolates examined and should not be generalized across S. aureus lineages. They do not establish direct SaeRS inhibition or reduction of virulence‐associated phenotypes because only four isolates were assessed and no extract‐only, Zn^2+^, chemically synthesized ZnO‐NP, downstream virulence, protein‐level, or functional controls were included. Larger controlled studies are required before biological or therapeutic implications can be drawn.

## 1. Introduction

*Staphylococcus aureus* is an adaptable clinical pathogen that causes infections ranging from superficial skin disease to sepsis, pneumonia, and infective endocarditis [1, 2]. Its pathogenicity depends on coordinated expression of adhesins, exoenzymes, and toxins in response to host and environmental signals. The *pvl* gene encodes Panton–Valentine leukocidin, a leukocyte‐targeting cytotoxin associated with tissue injury, whereas the accessory gene regulator (*agr*) quorum‐sensing system controls multiple virulence determinants through four major allelic groups (*agrI*–*agrIV*) [3].

Olive leaf extract contains phenolic compounds, including oleuropein, that can participate in the reduction and surface stabilization of zinc oxide nanoparticles (ZnO NPs) during plant‐mediated synthesis. This synthesis route reduces reliance on conventional chemical reducing agents and may influence nanoparticle surface chemistry [4].

The antibacterial activity of ZnO NPs has been attributed to several nonexclusive processes, including Zn^2+^ release, reactive oxygen species generation, and membrane damage [5, 6]. These processes can alter bacterial stress responses and transcription; however, a change in transcript abundance does not by itself demonstrate pathway‐specific inhibition. The SaeRS two‐component system regulates several *S.taphylococcus aureus* virulence determinants and therefore provides a relevant transcriptional target for assessing bacterial responses to subinhibitory nanoparticle exposure [7].

This study examined *saeR* and *saeS* expression after four multidrug‐resistant *Staphylococcus aureus* isolates were exposed to olive leaf–mediated ZnO NPs at 1/2 MIC. Conventional PCR was used to determine the prevalence of *pvl* and *agr* groups, and quantitative reverse transcription PCR (RT‐qPCR) was used to quantify *saeR* and *saeS* transcripts. The study was designed to identify an exploratory transcriptional association; it did not test direct binding to SaeRS, downstream protein activity, virulence phenotypes, or in vivo efficacy.

## 2. Materials and Methods

### 2.1. Collection and Identification of Bacterial Isolates

A total of 200 clinical specimens, including blood, wound‐derived samples, swabs, and urine specimens, were collected from patients admitted to Medical City Teaching Hospital, Baghdad, Iraq, between January and June 2024. Forty *Staphylococcus aureus* isolates were recovered. Preliminary identification was based on colony morphology on blood agar, Gram staining, and catalase and coagulase tests. Species‐level identification was confirmed using the VITEK 2 Compact system (bioMérieux, France). PCR amplification of the 16S rRNA gene was used as an additional molecular confirmation step.

### 2.2. Inclusion and Exclusion Criteria

Isolates were eligible if they were recovered from clinical specimens during the study period and confirmed as *Staphylococcus aureus* by the stated phenotypic and automated identification methods. Duplicate isolates from the same patient, contaminated cultures, and isolates with inconclusive identification were excluded.

### 2.3. Antimicrobial Susceptibility Testing

Antimicrobial susceptibility testing of *Staphylococcus aureus* isolates was performed using the Kirby–Bauer disk diffusion method according to Clinical and Laboratory Standards Institute (CLSI) standards [8, 9]. MRSA identification in this study was based on phenotypic resistance to oxacillin according to CLSI guidelines. Antibiotic disks were obtained from Liofilchem (Italy) and used at the following concentrations: gentamicin (10 μg), moxifloxacin (5 μg), clindamycin (2 μg), tigecycline (15 μg), erythromycin (15 μg), doxycycline (30 μg), ceftaroline (30 μg), rifampin (5 μg), fusidic acid (10 μg), vancomycin (30 μg), teicoplanin (30 μg), ciprofloxacin (5 μg), tetracycline (30 μg), trimethoprim–sulfamethoxazole (25 μg), chloramphenicol (30 μg), oxacillin (1 μg), and linezolid (30 μg). Isolates were classified as multidrug‐resistant when they were nonsusceptible to at least one agent in three or more antimicrobial categories [10].

### 2.4. DNA Extraction

Genomic DNA was extracted using the boiling method. A loopful of overnight bacterial culture was suspended in 1 mL of sterile distilled water, heated at 100°C for 10 min, rapidly cooled on ice, and centrifuged at 12,000 rpm for 5 min. The DNA‐containing supernatant was transferred to a sterile tube and stored at −20°C until PCR analysis [11].

### 2.5. Polymerase Chain Reaction (PCR)

Conventional PCR assays were performed to detect *pvl*, *agrI*, *agrII*, and *agrIII*. Amplification of the 16S rRNA gene served as a molecular confirmation and internal amplification control; it was not used to normalize conventional PCR signals. Primer sequences and expected amplicon sizes are provided in Table 1. Each 25 μL reaction contained 1 μL of forward primer, 1 μL of reverse primer, 7 μL of Master Mix (Amplicon, Denmark), 1 μL of DNA template, and 15 μL of double‐distilled water.

**TABLE 1:** Oligonucleotide primer sequences used for conventional PCR and RT‐qPCR analyses.

Gene	Nucleotide sequence (5′ ⟶ 3′)	Amplicon size (bp)	Reference
*Conventional PCR*			
*pvl*	Fw: ATCATTAGGTAAAATGTCTGGACATGATCCA	433	[16]
	Rv: GCATCAAGTGTATTGGATAGCAAAAGC		
*agrI*	Fw: ATGCACATGGTGCACATGC	441	[17]
	Rv: GTCACAAGTACTATAAGCTGCGAT		
*agrII*	Fw: ATGCACATGGTGCACATGC	575	
	Rv: TATTACTAATTGAAAAGTGGCCATAGC		
*agrIII*	Fw: ATGCACATGGTGCACATGC	323	
	Rv: GTAATGTAATAGCTTGTATAATAATACCCAG		

*RT-qPCR*			
*saeS*	Fw: CGTTATTTTGTTGCGCGAGT	340	This study
	Rv: CGTATCAAACATGAAAACCGCA		
*saeR*	Fw: ACGTAAAACGAGTTCCCTTGG	250	
	Rv: AACAACGACAACTAGCGGTAA		

*Reference gene*			
16S rRNA	Fw: CTACAATGGACAATACAAAGGGC	141	[18]
	Rv: TCACCGTAGCATGCTGATCT		

*Note:* Fw, forward primer; Rv, reverse primer.

Abbreviation: bp, base pairs.

Thermal cycling comprised initial denaturation at 94°C for 5 min, followed by 30 cycles of denaturation at 94°C for 60 s, primer‐dependent annealing at 58°C–60°C for 60 s, and extension at 72°C for 60 s, with a final extension at 72°C for 5 min.

PCR products were separated by electrophoresis on a 1.5% agarose gel and visualized under UV illumination using a gel documentation system [12]. A positive control containing previously confirmed *S. aureus* DNA and a negative control containing nuclease‐free water were included in each PCR run to ensure amplification accuracy and exclude contamination.

### 2.6. Effect of ZnO‐NP Exposure on Virulence‐Regulator Gene Expression

#### 2.6.1. Green Synthesis of ZnO NPs Using *Olea europaea* Leaf Extract

The ZnO nanoparticles were prepared as a single large‐scale batch using a standardized green synthesis protocol with olive leaf extract as the biological matrix. The same prepared batch was used for physicochemical characterization and subsequent biological experiments to minimize variability related to nanoparticle preparation. ZnO NPs were synthesized using olive leaf extract according to the method described by Al‐Momani et al. [13]; the recorded procedural steps used in the present study are summarized below. Fresh olive leaves collected in Baghdad, Iraq, were washed with double‐distilled water, dried at room temperature, and ground to a coarse powder.

Five grams of leaf powder was mixed with 50 mL of distilled water and heated in a water bath at 70°C for 15 min. The mixture was filtered, and zinc acetate dihydrate (Zn(CH_3_COO)_2_·2 H_2_O) was used as the zinc precursor. The extract was analyzed by gas chromatography–mass spectrometry to profile detectable constituents and was stored at −4°C until nanoparticle synthesis.

The same powder‐to‐water ratio, extraction temperature, extraction time, and solvent volume were used for each batch. The pH of the reaction mixture was adjusted to 10 with NaOH, and the mixture was stirred at room temperature for 2 h.

#### 2.6.2. Characterization of ZnO NPs

The characterization available in this study comprised X‐ray diffraction (XRD), scanning electron microscopy (SEM), and energy‐dispersive X‐ray spectroscopy (EDS), which provided crystallographic, morphological, and elemental information, respectively. Fourier‐transform infrared spectroscopy, transmission electron microscopy, hydrodynamic size distribution, polydispersity index, and zeta‐potential measurements were not available.

XRD patterns were acquired using a Shimadzu XRD‐6000 diffractometer to assess crystal phase [13]. XRD‐derived crystallite‐size estimates were obtained using X’Pert HighScore software.

Particle morphology and agglomeration were examined by SEM using a MIRA3 LMU instrument at magnifications from 2500 × to 1,000,00 × [13, 14]. Elemental composition was assessed by EDS [15].

#### 2.6.3. Minimum Inhibitory Concentration (MIC) and Sub‐MIC Determination

The MIC of the synthesized ZnO NPs was determined by broth microdilution in accordance with CLSI guidance [9]. Bacterial suspensions were exposed to serial twofold dilutions of ZnO NPs in Mueller–Hinton broth over a concentration range of 4–512 μg/mL.

Each well of a 96‐well microtiter plate was inoculated with a bacterial suspension adjusted to a 0.5 McFarland standard. Growth‐control wells contained inoculated broth without ZnO NPs, sterility‐control wells contained uninoculated broth, and treatment wells contained inoculated broth with the specified ZnO‐NP concentrations. Plates were incubated at 37°C for 18–24 h. The MIC was defined as the lowest ZnO‐NP concentration with no visible bacterial growth. For each isolate, 1/2 MIC was selected for subsequent gene‐expression analysis as a subinhibitory exposure condition to investigate transcriptional responses below the inhibitory threshold. MIC determinations were performed in triplicate. RT‐qPCR was conducted at this single exposure level; a multiconcentration transcriptional dose–response experiment was not performed.

#### 2.6.4. RNA Extraction and RT‐qPCR Analysis

Four MDR *Staphylococcus aureus* isolates were purposively selected from the larger collection of 40 isolates for exploratory RT‐qPCR analysis. The selected isolates exhibited the highest antimicrobial resistance profiles and carried different virulence‐associated gene profiles compared with the remaining isolates. Therefore, these isolates were selected as representative high‐risk MDR strains to investigate the transcriptional response of the SaeRS regulatory system following ZnO NP exposure. This pilot component was designed to characterize isolate‐level transcriptional response patterns of the saeRS two‐component regulatory system following ZnO NP exposure rather than to provide population‐level inference.

Cultures were exposed to green‐synthesized ZnO NPs at 1/2 MIC before RNA extraction. For each isolate, an untreated culture processed in parallel served as the matched calibrator for relative gene‐expression analysis.

Total RNA was extracted using a commercial RNA extraction kit (Geneaid, Taiwan) according to the manufacturer’s instructions. RNA quantity and purity were assessed using a NanoDrop spectrophotometer at 260 and 280 nm; samples with A260/A280 ratios of approximately 1.8–2.0 were used for downstream analysis. RNA was treated with DNase before complementary DNA (cDNA) synthesis. cDNA was synthesized separately from 1 μg of total RNA using a reverse‐transcription kit (Bioneer, South Korea) according to the manufacturer’s protocol. A no‐reverse‐transcription control was included to verify the absence of amplifiable genomic DNA.

RT‐qPCR was performed using a CFX96 Touch Real‐Time PCR Detection System (Bio‐Rad, USA). Each 20 μL reaction contained SYBR Green Master Mix (Promega, USA), 0.2 μM of each forward and reverse primer, and 2 μL of diluted cDNA template. The 16S rRNA gene was selected as the internal reference because of its extensive use in bacterial RT‐qPCR studies, including *Staphylococcus aureus*, and its suitability for comparative expression analysis under controlled experimental conditions. Primer sequences and expected amplicon sizes are listed in Table 1. Primer specificity and amplification efficiency were evaluated using standard curves generated from serial cDNA dilutions; all primer sets showed efficiencies of 90%–110%.

The thermal program comprised initial denaturation at 95°C for 5 min, followed by 40 cycles of denaturation at 95°C for 20 s, annealing at 54°C for 20 s, and extension at 72°C for 20 s. A no‐template control was included in each run, and each biological sample was analyzed in technical triplicate. Melt‐curve analysis from 65°C to 95°C was performed after amplification to confirm product specificity and exclude nonspecific products or primer‐dimer formation.

Relative expression of *saeR* and *saeS* was calculated using the paired comparative‐threshold‐cycle method described by Livak and Schmittgen [19]. The 16S rRNA gene was used as the internal reference, and the untreated culture corresponding to each isolate was used as its matched calibrator.

(1) ΔCti=Cttarget, i−Ct16S rRNA, i,

(2) ΔΔCti=ΔCttreated, i−ΔCtuntreated, i,

(3) Relative expressioni=2−ΔΔCti.

A relative‐expression value below 1 indicated lower target‐transcript abundance in the treated culture than in its matched untreated control, whereas a value above 1 indicated higher expression. Statistical testing was performed on paired ΔCt values rather than on transformed fold‐change values. Because only four isolate pairs were examined at one sub‐MIC concentration, the fold‐change estimates were interpreted as descriptive pilot observations.

### 2.7. Ethical Approval

This study was conducted in accordance with the ethical principles of biomedical research involving human‐derived specimens. Clinical specimens were collected between January and June 2024 as part of routine diagnostic procedures at Medical City Teaching Hospital, Baghdad, Iraq. Written informed consent was obtained from all participants prior to sample collection. All specimens were anonymized before laboratory analysis, and no personal identifying information was recorded. The study protocol was reviewed and approved by the Institutional Ethics Committee of Mustansiriyah University, Baghdad, Iraq, on December 1, 2023 (Approval No.: BCSMU/1024/00086M).

### 2.8. Statistical Analysis

Statistical analyses were performed in R Version 4.5.0. Gene prevalence was summarized as counts and percentages without an inferential comparison among *pvl* and *agr* detection frequencies because these outcomes were measured within the same isolate set and were not independent categories. For RT‐qPCR, the four clinical isolates constituted four matched biological pairs (*n* = 4); technical triplicates were used to assess assay repeatability and were not treated as independent observations. For each gene, untreated and 1/2‐MIC‐exposed ΔCt values from the same isolate were compared using an exact two‐sided Wilcoxon signed‐rank test. Testing was conducted on ΔCt values because fold‐change values are nonlinearly transformed. Exact *p* values are reported, and *p* < 0.05 was considered statistically significant. Given the small number of biological pairs and the limited resolution of the exact test, all inferential findings were interpreted as exploratory.

## 3. Results

### 3.1. Characterization of ZnO NPs

The XRD pattern contained its highest‐intensity peak at 36.30° 2*θ* (d‐spacing, 2.475 Å), with additional prominent peaks at 31.82° and 34.47° 2*θ* (d‐spacings, 2.812 and 2.602 Å, respectively). The observed diffraction pattern was consistent with crystalline ZnO formation (Figure 1).

**FIGURE 1:**
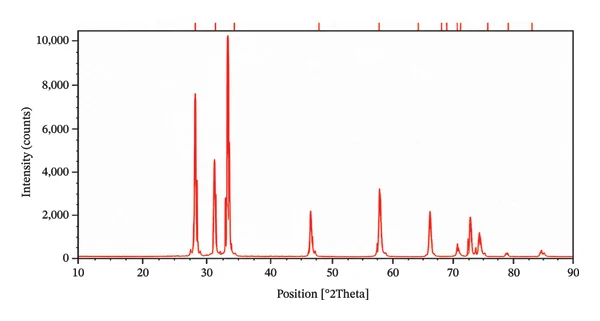
XRD pattern of the synthesized ZnO nanoparticles. The *x*‐axis represents the diffraction angle (2θ), and the *y*‐axis represents diffraction intensity.

XRD‐derived crystallite‐size estimates ranged from 5.2 to 99.3 nm. Most estimates were between 5 and 20 nm; the mean was 19.24 nm with a standard deviation of 18.25 nm (Figure 2).

**FIGURE 2:**
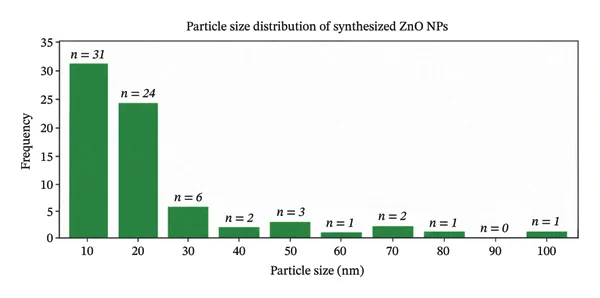
XRD‐derived crystallite size distribution of the synthesized ZnO nanoparticles.

SEM images presented in Figure 3 showed aggregates composed of smaller, approximately rounded particles. Agglomeration was evident across the examined magnifications, with finer particle features visible at higher magnification.

**FIGURE 3:**
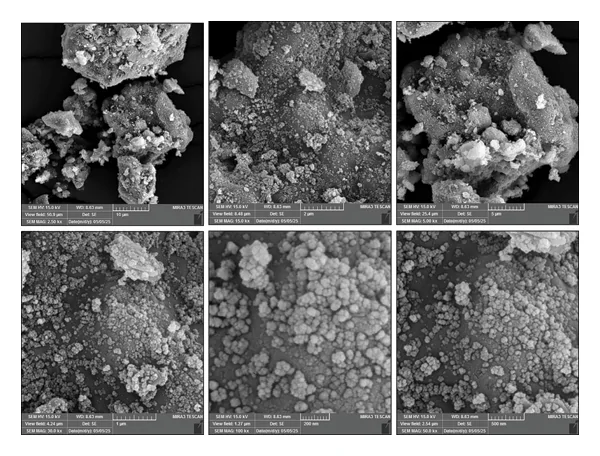
Scanning electron microscopy images showing the morphology and agglomeration of green‐synthesized ZnO nanoparticles at magnifications from 2500 × to 1,000,00×.

The EDS spectrum presented in Figure 4 showed a dominant zinc signal near 1.0 keV, additional zinc signals near 8.6 and 9.6 keV, and an oxygen signal near 0.5 keV. The reported elemental composition was 84.24% zinc and 15.76% oxygen. The spectrum supports the presence of Zn and O but does not, by itself, establish chemical purity.

**FIGURE 4:**
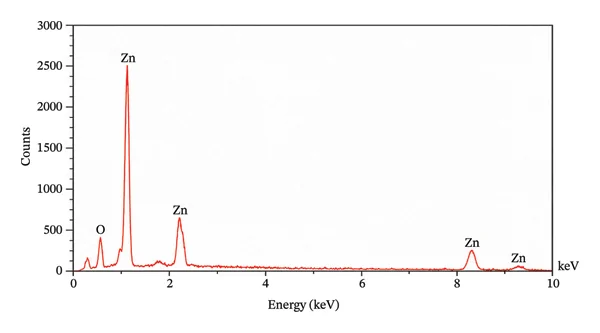
Energy‐dispersive X‐ray spectrum of the synthesized ZnO nanoparticles, showing the principal zinc and oxygen signals.

### 3.2. Antimicrobial Resistance Patterns Among Clinical Isolates

Among the 40 clinical *Staphylococcus aureus* isolates, resistance was highest to oxacillin (85.0%), fusidic acid (82.5%), ceftaroline (72.5%), and rifampin (70.0%). Resistance to gentamicin, doxycycline, tigecycline, and linezolid was 2.5% for each agent. These results describe in vitro susceptibility within the sampled isolate set and should not be interpreted as comparative clinical efficacy. Full susceptibility results are presented in Table 2.

**TABLE 2:** Antimicrobial susceptibility patterns of the clinical isolates.

Antibiotic class	Antibiotic	Sensitive isolates (*n*, %)	Resistant isolates (*n*, %)
Aminoglycosides	Gentamicin	39 (97.5%)	1 (2.5%)
Fluoroquinolones	Moxifloxacin	31 (77.5%)	9 (22.5%)
Fluoroquinolones	Ciprofloxacin	35 (87.5%)	5 (12.5%)
Macrolides	Erythromycin	9 (22.5%)	31 (77.5%)
Lincosamides	Clindamycin	33 (82.5%)	7 (17.5%)
Tetracyclines	Tigecycline	39 (97.5%)	1 (2.5%)
Tetracyclines	Doxycycline	39 (97.5%)	1 (2.5%)
Tetracyclines	Tetracycline	34 (85.0%)	6 (15.0%)
Glycopeptides	Vancomycin	38 (95.0%)	2 (5.0%)
Glycopeptides	Teicoplanin	36 (90.0%)	4 (10.0%)
Oxazolidinones	Linezolid	39 (97.5%)	1 (2.5%)
Penicillins/cephalosporins	Ceftaroline	11 (27.5%)	29 (72.5%)
Penicillins/cephalosporins	Oxacillin	6 (15.0%)	34 (85.0%)
Rifamycins	Rifampin	12 (30.0%)	28 (70.0%)
Steroid antibiotics	Fusidic acid	7 (17.5%)	33 (82.5%)
Sulfonamides (combination)	Trimethoprim–sulfamethoxazole	35 (87.5%)	5 (12.5%)
Amphenicols	Chloramphenicol	32 (80.0%)	8 (20.0%)

### 3.3. Prevalence of Virulence‐Associated Genes

#### 3.3.1. Prevalence of *pvl* and *agr Genes* Among Clinical *Staphylococcus aureus* Isolates

Conventional PCR detected *pvl* in 27.5% (11/40) of isolates, *agrI* in 17.5% (7/40), *agrII* in 20.0% (8/40), and *agrIII* in 15.0% (6/40). These frequencies are presented descriptively in Figure 5.

**FIGURE 5:**
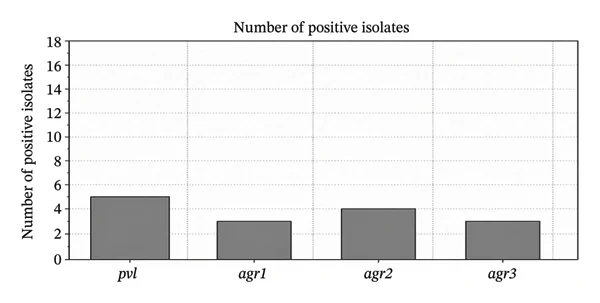
Numbers and percentages of *Staphylococcus aureus* isolates positive for *pvl* and *agr* groups I–III among the 40 isolates examined.

#### 3.3.2. SaeRS Gene Expression Following Sub‐MIC ZnO‐NP Exposure in *Staphylococcus aureus*

Paired ΔCt analysis showed a numerical reduction in *saeR* transcript abundance in all four isolates after exposure to ZnO NPs at 1/2 MIC; however, the exact two‐sided Wilcoxon signed‐rank test was not statistically significant (*p* = 0.125). Paired *saeR* ΔΔCt values ranged from 0.99 to 19.55, and the corresponding relative‐expression values ranged from 1.30 × 10^−6^ to 0.503 (median, 0.0102). For *saeS*, transcript abundance decreased in T1, T2, and T4 but increased in T3. This paired comparison was also not significant (*p* = 0.250). *saeS* relative‐expression values ranged from 1.58 × 10^−6^ to 1.682 (median, 0.000617). Neither gene met the prespecified significance threshold of 0.05, as shown in Figures 6 and 7 and summarized in Table 3.

**FIGURE 6:**
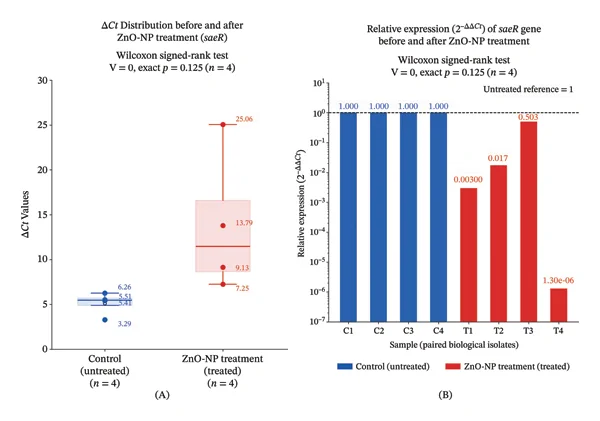
Expression of *saeR* in four matched multidrug‐resistant *Staphylococcus aureus* isolates before and after exposure to ZnO NPs at 1/2 MIC. Panel (A) shows the paired ΔCt distributions for untreated and treated cultures. Panel (B) shows isolate‐specific relative expression calculated as 2^−ΔΔCt^. The exact two‐sided Wilcoxon signed‐rank test yielded V = 0 and *p* = 0.125 (*n* = 4). Technical triplicates were not treated as independent biological observations.

**FIGURE 7:**
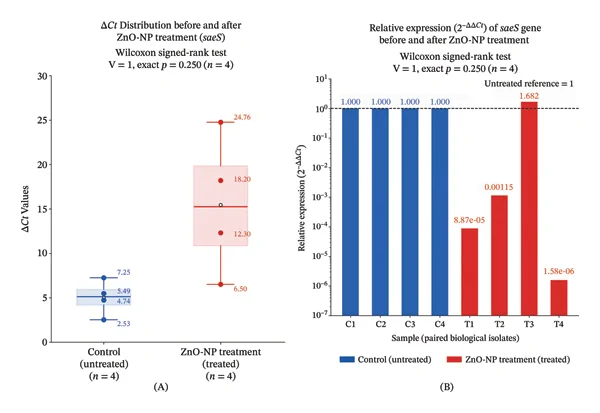
Expression of *saeS* in four matched multidrug‐resistant *Staphylococcus aureus* isolates before and after exposure to ZnO NPs at 1/2 MIC. Panel (A) shows the paired ΔCt distributions for untreated and treated cultures. Panel (B) shows isolate‐specific relative expression calculated as 2^−ΔΔCt^. The exact two‐sided Wilcoxon signed‐rank test yielded V = 1 and *p* = 0.250 (*n* = 4). Technical triplicates were not treated as independent biological observations.

**TABLE 3:** Paired isolate‐level RT‐qPCR calculations for *saeR* and *saeS*.

Gene	Matched pair	Untreated ΔCt	Exposed ΔCt	ΔΔCt	2^−^ΔΔCt	Direction
saeR	C1/T1	5.41	13.79	8.38	3.01 × 10^−3^	Lower
saeR	C2/T2	3.29	9.13	5.84	1.75 × 10^−2^	Lower
saeR	C3/T3	6.26	7.25	0.99	0.503	Lower
saeR	C4/T4	5.51	25.06	19.55	1.30 × 10^−6^	Lower
saeS	C1/T1	4.74	18.20	13.46	8.87 × 10^−5^	Lower
saeS	C2/T2	2.53	12.30	9.77	1.15 × 10^−3^	Lower
saeS	C3/T3	7.25	6.50	−0.75	1.682	Higher
saeS	C4/T4	5.49	24.76	19.27	1.58 × 10^−6^	Lower

*Note:* Each treated culture was compared with the untreated culture from the same isolate. Relative expression was calculated as 2^−^ΔΔCt. Values below 1 indicate lower expression after exposure.

The paired ΔΔCt and log_2_ relative‐expression profiles varied markedly among isolates (Figures 8 and 9). T1 and T4 showed large numerical decreases for both genes, whereas T3 showed a smaller decrease in *saeR* and numerical upregulation of *saeS*. These isolate‐level measurements are descriptive because only four biological pairs were available.

**FIGURE 8:**
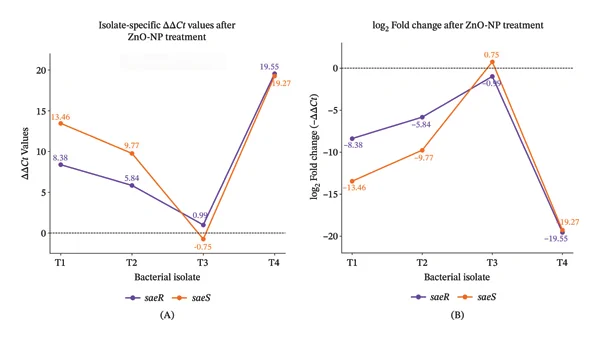
Isolate‐specific transcriptional responses of *saeR* and *saeS* after exposure to ZnO NPs at 1/2 MIC. Panel (A) presents paired ΔΔCt values, and Panel (B) presents the corresponding log_2_ fold changes (−ΔΔCt). Positive ΔΔCt values indicate lower expression after exposure, whereas a negative ΔΔCt value indicates higher expression relative to the matched untreated culture.

**FIGURE 9:**
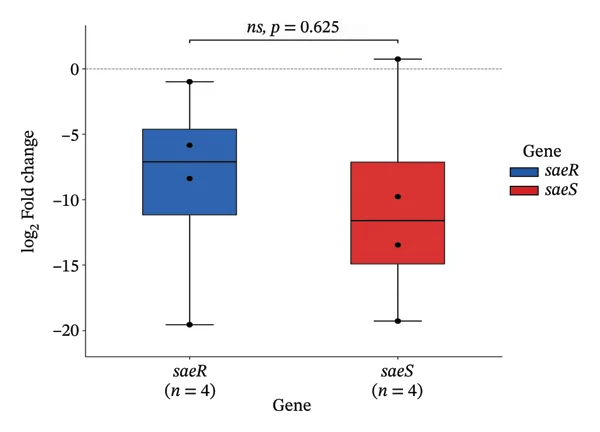
Descriptive comparison of isolate‐level log_2_ fold changes for *saeR* and *saeS*. Individual points represent the four matched isolates, and boxplots summarize their distributions. The within‐isolate exact two‐sided Wilcoxon signed‐rank comparison was not statistically significant (*p* = 0.625).

A within‐isolate comparison of *saeR* and *saeS* log_2_ relative‐expression responses did not show a statistically significant difference between the genes (exact two‐sided Wilcoxon signed‐rank *p* = 0.625). This exploratory comparison does not establish preferential modulation of either SaeRS component.

## 4. Discussion

Olive leaf extract was used as the biological matrix for ZnO‐NP synthesis; its independent antibacterial effect was not tested. Previous studies have reported antibacterial activity of olive‐derived phenolics and aqueous extracts [20, 21], but those findings cannot assign the present transcriptional response to the extract component. Because an extract‐only control was not included, the relative contributions of residual phytochemicals and the ZnO nanomaterial remain unresolved.

*Staphylococcus aureus* is a frequent cause of healthcare‐associated infection, and multidrug resistance narrows available treatment options [2]. Its pathogenicity also depends on regulatory networks that coordinate toxin production, immune evasion, adhesion, and biofilm‐associated traits. SaeRS is one such two‐component system; however, *saeR* and *saeS* transcript abundance represents only one level of this regulatory network.

XRD, SEM, and EDS supported the formation of crystalline, zinc‐ and oxygen‐containing material with aggregated, approximately rounded nanoscale features. Published studies describe reactive oxygen species, membrane perturbation, and Zn^2+^ release as nonexclusive contributors to ZnO‐NP antibacterial activity [5, 6]. None of these processes was directly quantified in the present study; therefore, they remain literature‐supported hypotheses rather than demonstrated mechanisms for the observed transcriptional changes.

The SEM images obtained in this study characterize the nanoparticles alone; they do not visualize attachment to bacterial cells or membrane damage. The data therefore cannot determine whether the *saeR*/*saeS* response resulted from reactive oxygen species, Zn^2+^ release, membrane perturbation, or another sublethal stress pathway. Direct mechanistic attribution would require bacterial imaging, reactive‐oxygen‐species measurements, zinc‐release analysis, and pathway‐specific functional experiments.

The isolate set showed high resistance to oxacillin, fusidic acid, ceftaroline, and rifampin. These rates document the phenotype of the sampled isolates; however, antimicrobial exposure histories, prescribing patterns, clonal relationships, and resistance‐gene profiles were not evaluated. The causes of the observed resistance pattern therefore cannot be inferred from the present data.

The *pvl* gene was detected in 27.5% of isolates, while *agrI*, *agrII*, and *agrIII* occurred at different frequencies. These results are broadly compatible with the genetic heterogeneity reported among clinical *Staphylococcus aureus* populations [22, 23]. Detection of these genes indicates genotype prevalence but does not demonstrate toxin production, *agr* activity, or clinical virulence in the sampled isolates.

At the transcriptional level, *saeR* ΔCt increased after exposure in all four isolate pairs, indicating a consistent numerical direction toward lower relative expression. Nevertheless, the exact paired comparison was not statistically significant (*p* = 0.125). *saeS* showed lower expression in three isolates and higher expression in T3 (*p* = 0.250). The findings therefore represent descriptive, isolate‐specific transcriptional responses rather than statistically significant downregulation of either gene. Given that the analysis was based on only four matched isolate pairs, these observations should be interpreted within the tested isolates and should not be generalized across genetically diverse *Staphylococcus aureus* lineages.

Because the present study focused on transcriptional responses following sub‐MIC ZnO NP exposure, direct assessment of bacterial viability after treatment was not included in the current experimental design. Future studies incorporating viability assays, such as viable colony counting or bacterial recovery analysis, will further confirm that observed transcriptional changes occur in surviving bacterial populations rather than reflecting reduced bacterial abundance. SaeRS regulates multiple virulence‐associated determinants; however, the present study assessed only *saeR* and *saeS* transcript levels [7]. Therefore, the observed transcriptional changes indicate modulation at the mRNA level but do not demonstrate direct inhibition of SaeRS pathway activity. Confirmation of pathway inhibition would require additional analyses, including SaeR/S protein abundance, phosphorylation status, downstream target gene expression, and functional virulence assays.

The magnitude and direction of the transcriptional response varied among isolates. T1 and T4 showed the largest numerical decreases for both genes, whereas T3 showed a modest decrease in *saeR* and an increase in *saeS*. Differences in genetic background, baseline SaeRS activity, nanoparticle–cell interactions, and stress adaptation could contribute to this heterogeneity, but these explanations were not experimentally distinguished. The small isolate panel and absence of a concentration–response design preclude a uniform interpretation.

SaeRS is connected with adhesion, toxin regulation, and biofilm‐associated processes, but biofilm formation was not measured in this study. The present data therefore do not demonstrate reduced biofilm formation or reduced virulence. Recent evidence from Baghdad reported high frequencies of *icaB*, *icaC*, *crtM*, and *mecA* among clinical *Staphylococcus aureus* isolates, while *mecA* prevalence was not significantly associated with stronger biofilm formation [24]. Any relationship between SaeRS transcription and biofilm behavior requires direct testing using validated biofilm assays, downstream transcriptional targets, protein‐level measurements, and appropriate functional models.

## 5. Limitations

The RT‐qPCR analysis was designed as an exploratory pilot assessment and was limited to four matched multidrug‐resistant clinical isolate pairs. This sample size restricts statistical power and generalizability. With four nonzero paired differences, the smallest attainable exact two‐sided Wilcoxon signed‐rank *p* value is 0.125; therefore, even the same‐direction *saeR* changes across all four isolates could not meet a 0.05 threshold. The gene‐expression findings should consequently be interpreted as descriptive pilot observations confined to the tested isolates and exposure conditions. Only one sub‐MIC exposure concentration (1/2 MIC) was examined, and the RT‐qPCR evaluation included a limited number of biological isolates. The study did not include olive‐leaf‐extract‐only, soluble Zn^2+^, or chemically synthesized ZnO‐NP controls. Reactive oxygen species, zinc release, bacterial viability after sub‐MIC exposure, membrane damage, downstream virulence genes, protein abundance, biofilm formation, and virulence phenotypes were not measured. The 16S rRNA gene was selected as the normalization reference based on its widespread application in bacterial transcriptional studies. However, validation using additional housekeeping genes, such as gyrB or rpoB, may further strengthen normalization accuracy in future ZnO‐NP stress‐response studies. The study therefore cannot distinguish nanoparticle‐specific modulation of virulence‐associated regulation from a broader sublethal stress response. Future expanded studies should incorporate multiple sub‐MIC concentrations and larger collections of genetically diverse *Staphylococcus aureus* isolates to evaluate concentration‐dependent transcriptional responses, improve reproducibility, and strengthen the generalizability of the findings. Nanoparticle characterization was limited to XRD, SEM, and EDS. Fourier‐transform infrared spectroscopy, transmission electron microscopy, hydrodynamic size, polydispersity, and zeta potential were unavailable, limiting assessment of surface chemistry and colloidal stability. Together with the small isolate panel and restricted control design, these constraints limit functional validation, mechanistic depth, and causal interpretation.

## 6. Conclusion

ZnO NP exposure was associated with altered *saeR* and *saeS* transcriptional patterns under sub‐MIC conditions among the tested MDR *S. aureus* isolates. Specifically, *saeR* showed a consistent numerical reduction across all four isolates, although the paired comparison was not statistically significant (*p* = 0.125), while *saeS* expression decreased in three isolates and increased in one isolate (*p* = 0.250). These findings represent descriptive, isolate‐specific transcriptional responses rather than statistically significant downregulation of either gene. Given the four matched biological pairs analyzed, these observations should not be generalized across genetically diverse *Staphylococcus aureus* lineages. The consistent numerical reduction observed for *saeR* across all tested isolates indicates a transcriptional pattern that warrants further investigation in larger isolate collections. The findings do not demonstrate inhibition of SaeRS regulatory activity, reduced toxin production, anti‐biofilm activity, or therapeutic efficacy. The absence of component‐specific controls prevents attribution of the response specifically to ZnO nanoparticles rather than residual olive‐derived compounds or soluble zinc. Further studies should include larger genetically diverse isolate panels, multiple subinhibitory concentrations, appropriate extract and ZnO controls, expanded physicochemical characterization, and downstream functional assays to determine reproducibility and biological relevance.

## Funding

No funding was received for this manuscript.

## Conflicts of Interest

The authors declare no conflicts of interest.

## Data Availability

The data that support the findings of this study are available on request from the corresponding author. The data are not publicly available due to privacy or ethical restrictions.
